# Effects of Dietary Mannan Oligosaccharides on Non-Specific Immunity, Intestinal Health, and Antibiotic Resistance Genes in Pacific White Shrimp *Litopenaeus vannamei*

**DOI:** 10.3389/fimmu.2021.772570

**Published:** 2021-11-24

**Authors:** Tiantian Wang, Jinzhu Yang, Gang Lin, Mingzhu Li, Ronghua Zhu, Yanjiao Zhang, Kangsen Mai

**Affiliations:** ^1^ The Key Laboratory of Aquaculture Nutrition and Feed (Ministry of Agriculture), The Key Laboratory of Mariculture (Ministry of Education), Ocean University of China, Qingdao, China; ^2^ Institute of Quality Standards and Testing Technology for Agricultural Products, Chinese Academy of Agricultural Sciences, Beijing, China; ^3^ College of Agriculture, Ludong University, Yantai, China; ^4^ Beijing Alltech Biological Products (China) Co., Ltd., Beijing, China

**Keywords:** mannan oligosaccharide, immunity, intestinal microbiota, antibiotic resistance genes, *Litopenaeus vannamei*

## Abstract

This study was conducted to comprehensively investigate the beneficial effects of a mannan oligosaccharide product (hereinafter called MOS) on *Litopenaeus vannamei* and optimum level of MOS. Five isonitrogenous and isolipid diets were formulated by adding 0%, 0.02%, 0.04%, 0.08%, and 0.16% MOS in the basal diet. Each diet was randomly fed to one group with four replicates of shrimp in an 8-week feeding trial. The results showed that dietary MOS improved the growth performance and the ability of digestion of shrimp. Dietary MOS significantly increased the activity of total superoxide dismutase, catalase, and glutathione peroxidase and decreased the content of malondialdehyde in plasma of shrimp. Dietary MOS significantly increased the activity of alkaline phosphatase and lysozyme in plasma and the hemocyte counts. Dietary MOS significantly upregulated the expression of Toll, lysozyme, anti-lipopolysaccharide factor, Crustin, and heat shock protein 70 in the hepatopancreas. And dietary MOS significantly upregulated the expression of intestinal mucin-2, mucin-5B, and mucin-19, while it decreased the expression of intestinal mucin-1 and macrophage migration inhibitory factor. Dietary MOS improved the bacterial diversity; increased the abundance of *Lactobacillus*, *Bifidobacterium*, *Blautia*, and *Pseudoalteromonas*; and decreased the abundance of *Vibrio* in the intestine. Shrimp fed MOS diets showed lower mortality after being challenged by *Vibrio parahaemolyticus*. Notably, this study found a decrease in antibiotic resistance genes and mobile genetic elements after MOS supplementation for the first time. The present results showed that diet with MOS supplementation enhanced the organismal antioxidant capacity and immunity, improved intestinal immunity, optimized intestinal microecology, mitigated the degree of antibiotic resistance, and increased the resistance to *V. parahaemolyticus* in *L. vannamei*, especially when supplemented at 0.08% and 0.16%.

## Introduction

Pacific white shrimp (*Litopenaeus vannamei*) have great economic value and are widely farmed throughout the world due to their nutritional value, rapid growth, and high capacity to adapt to the environment (1, 2). However, poor environmental conditions and inappropriate management practices in intensive aquaculture have resulted in reduced immunity and frequent diseases (3). For the past decades, antibiotics were widely used in the control of diseases in livestock, poultry, and aquatic animals. However, the application of antibiotics was under criticism in consideration of antibiotic resistance, environmental hazards, and accumulation of residues in seafood and subsequently human tissues (4). Therefore, it is imperative to search for environment-friendly and safe alternative method to improve the health of shrimp.

In recent years, there has been an increasing interest in promoting animal health through functional feed additives. Mannan oligosaccharide (MOS), derived from the cell wall of *Saccharomyces cerevisiae*, has been recently used in aquaculture. Without being digested by digestive enzyme, MOS could reach the distal intestine, where it was selectively fermented by intestinal microorganisms and decomposed into organic acid, carbon dioxide, or hydrogen (5, 6). The main beneficial effects of MOS were fish performance elevation by stimulation of the innate immune system and improvement of gut functions and nutrient digestibility, partly through reducing colonization of pathogen in the gastrointestinal tract (7–10). The positive effects of MOS supplementation in aqua feed have been reported in various fish species such as rainbow trout (*Oncorhynchus mykiss*) (11, 12), channel catfish (*Ictalurus punctatus*) (13), turbot (*Scophthalmus maximus* L.) (14), European sea bass (*Dicentrarchus labrax*) (15–17), and hybrid grouper (*Epinephelus lanceolatus* ♂ × *Epinephelus fuscoguttatus* ♀) (18). Studies on shrimp have shown that the addition of MOS in diets was conducive to weight gain, immunity (1, 19–21), and digestive enzyme activity (22). The intestine plays a crucial role in nutrient digestion and absorption as well as disease defense in shrimp and is responsible for nearly 70% of the immune function of shrimp. However, few studies have been conducted to investigate the effects of MOS on shrimp intestinal health.

MOS used in this experiment (Actigen^™^) was a prebiotic product with higher MOS purity and activity, purified from *S. cerevisiae* 1026 using nutrigenomics technology. Especially, a mannan-rich fraction from *S. cerevisiae* reduced the growth rate and positively influenced the antibiotic susceptibility of resistant *Enterobacteria* (23, 24). Antibiotic resistance genes (ARGs) are the basic cause of antibiotic resistance. However, research on the effect of MOSs on ARGs is currently very limited. At present, research on bacterial resistance to antibiotics was mostly based on microbial cultivation (25–27); however, there are a large number of unculturable microorganisms in the environment, making this method not comprehensive. By contrast, high-throughput qPCR (HT-qPCR) obtains qPCR chips by packaging primers for 383 resistance genes on a chip and is consequently considered to be efficient, high throughput, highly accurate, and sensitive for multiple samples (28).

To this end, different levels of MOS were supplemented into basal diets to investigate the effects of MOS on growth performance, immunity, intestinal immunity, intestinal microbiota, and ARGs of *L. vannamei*. The results could contribute to healthy cultivation of *L. vannamei*.

## Materials and Methods

### Experimental Diets

The MOS product (Actigen™, item number: 07.2301.067.087.CN0.36) used in this experiment was provided by Beijing Alltech Biological Products Co., Ltd. (China) (with 12% MOS in product, hereinafter called MOS). Five experimental diets were formulated to contain different levels of MOS at 0%, 0.02%, 0.04%, 0.08%, and 0.16% (designated as M0, M2, M4, M8, and M16). All ingredients were thoroughly mixed with fish oil, soybean oil, and krill oil; and then water was added to produce a stiff dough. The dough was then pelleted and dried until constant weight in a ventilated oven at 55°C and stored in a freezer at −20°C until use. The chemical composition of diets is shown in **Table 1**.

**Table 1:** Formulation and proximate composition of the experimental diets (% dry matter).

Ingredients (%)	Diets				
	M0	M2	M4	M8	M16
Fish meal	15.00	15.00	15.00	15.00	15.00
Shrimp meal	5.00	5.00	5.00	5.00	5.00
Beer yeast powder	5.00	5.00	5.00	5.00	5.00
Soybean meal	30.00	30.00	30.00	30.00	30.00
Dephenolized cottonseed protein	5.00	5.00	5.00	5.00	5.00
Peanut meal	10.00	10.00	10.00	10.00	10.00
Wheat flour	22.00	22.00	22.00	22.00	22.00
Fish oil	1.00	1.00	1.00	1.00	1.00
Soybean oil	1.00	1.00	1.00	1.00	1.00
Lecithin oil	1.00	1.00	1.00	1.00	1.00
Monocalcium phosphate	1.00	1.00	1.00	1.00	1.00
Choline chloride	0.20	0.20	0.20	0.20	0.20
Vitamin premix ^1^	0.20	0.20	0.20	0.20	0.20
Mineral premix ^2^	1.00	1.00	1.00	1.00	1.00
Lysine hydrochloride	0.10	0.10	0.10	0.10	0.10
Solid methionine	0.10	0.10	0.10	0.10	0.10
Threonine	0.05	0.05	0.05	0.05	0.05
Vitamin C	0.20	0.20	0.20	0.20	0.20
MOS	0	0.02	0.04	0.08	0.16
Rice bran meal	2.15	2.13	2.11	2.07	1.99
Analyzed nutrients compositions (% dry matter)					
Crude protein	43.64	43.28	44.41	44.07	43.00
Crude lipid	5.75	5.92	5.73	5.77	6.07
Crude ash	9.06	9.10	8.73	9.07	9.01

MOS, mannan oligosaccharide.

^1^Per kg vitamin premix contains: vitamin A acetate, 714,000 IU; vitamin D3, 266,000 IU; dl-α-tocopherol acetate, 8.6 g; menadione, 1 g; thiamine mononitrate, 1 g; riboflavin, 1.4 g; pyridoxine hydrochloride, 1.2 g; cyanocobalamin, 0.004 g; calcium d-pantothenate, 4 g; nicotinamide, 6.8 g; folic acid, 0.28 g; d-biotin, 0.012 g; inositol, 7.6 g; l-ascorbic acid-2-phosphate, 16.6 g.

^2^Per kg mineral premix contains: Mg, 12.5 g; Zn, 7.5 g; Mn, 2 g; Cu, 1.25 g; Co, 0.05 g; Se, 0.015 g; I, 0.05 g.

Proximate composition analysis of feed ingredients and diets were analyzed following the standard methods (AOAC, 1995). Moisture content was determined by drying samples to a constant weight at 105°C; crude protein by Kjeldahl method (FOSS 8400, Sweden); crude lipid by Soxhlet method (BUCHI 36880, Switzerland); and crude ash content by combustion at 550°C.

### Feeding Trial

The experiment was conducted at Huanghai Aquatic Co. Ltd. (China), and Pacific white shrimp (*L. vannamei*) were purchased from a local commercial farm. Prior to the start of feeding trial, shrimp were fed with the control diet for 2 weeks to acclimate the experimental environment. Then a total of 800 shrimp (initial mean weight of 2.40 ± 0.03 g) were randomly distributed into 20 tanks (square, 200 L) with 40 shrimp per tank. Experimental diets were fed to shrimp four times daily (05:30, 11:00, 16:30, and 21:30) for 8 weeks. The daily feeding quantity was 6%–8% of body weight and adjusted according to previous feeding response. The seawater was transferred into a reservoir for sedimentation before being pumped into the farming system, and two-thirds volume per tank was exchanged twice daily. During the feeding trial, the temperature was 26.4°C–28.0°C; salinity was 31‰–33‰; pH was 8.2–8.4; dissolved oxygen was higher than 7 mg/L; nitrite was lower than 0.005 mg/L; nitrate was lower than 15 mg/L; and ammonia was lower than 0.02 mg/L.

### Sample Collection

At the end of the feeding trial, shrimp were fasted for 24 h, and then the body length and weight of each shrimp were measured. Hemolymph of 12 shrimp per tank was obtained using a 1-ml sterile syringe and diluted immediately at a ratio of 1:1.5 of hemolymph to anticoagulant (10 mmol/L of EDTA-Na_2_, 450 mmol/L of NaCl, 10 mmol/L of KCl, and 10 mmol/L of HEPES, at pH 7.3). A fraction was used for hemocyte count. The rest was centrifuged at 500 g for 10 min at 4°C, and then the supernatant was collected and stored at −80°C. The hepatopancreas were quickly removed from aforesaid shrimp and then transferred to a 1.5-ml sterile RNase-free centrifuge tube (Axygen, USA) for analysis of enzyme activity and gene expression. Then the intestines were removed and transferred to 2-ml sterile tubes (Axygen, USA), of which six were used for gene expression analysis and the other six were used for 16S rRNA and ARGs analysis. The above hepatopancreas and intestines were frozen in liquid nitrogen once dissected and then stored at −80°C. The muscle of four fresh shrimp from each tank was collected and stored at −20°C for analysis of biochemical composition.

### Growth Performance

Growth performance was calculated by using the following variables:

$Weight\;gain\;rate\;\left(WGR;\;\%\right)\;=\;100\;\times \;(W_{2}-W_{1})/W_{1}$

$Specific\;growth\;rate\;\left(SGR;\;\%/day\right)\;=\;100\;\times \;\left(Ln\;W_{2}-\;Ln\;W_{1}\right)/days$ $Feed\;intake\;\left(FI;\;\%/day\right)\;=\;100\;\times D/\left[\left(W_{1}+W_{2}\right)/2\right]/days$

$Feed\;efficiency\;\left(FE\right)\;=\;\left(W_{2}–W_{1}\right)/D$

*W*_1_ is the initial body weight; *W*_2_, final body weight; and *D*, total amount of feed consumptions.

### Hemocyte Count

The hemocyte count was measured with hemocytometer under light microscope (Nikon, E 600, Japan). The number of blood cells per ml of hemolymph was calculated according to the formula below:

Hemocyte count in 1 ml of hemolymph =A/5 × 25 × 10,000 × B

*A* is the total hemocyte count in five medium squares and *B* is the dilution ratio of the sample.

### Enzyme Activity

The hepatopancreas and intestines were weighted, thawed, and homogenized (1:9) in ice-cold 0.9% NaCl solution (pH 7). After centrifugation (2,500 rpm, 10 min, 4°C), the supernatant was collected and stored at −80°C until analysis. Total antioxidation capability (T-AOC; A015-2-1), malondialdehyde (MDA; A003-1-2), total superoxide dismutase (TSOD; A001-1), catalase (CAT; A007-1-1), and glutathione peroxidase (GPX; A005-1-2) activity in plasma were determined with assay kits (Nanjing Jiancheng Bioengineering Institute, Nanjing, China). Phenol oxidase (PO) activity (H247) was determined using ELISA kit (Nanjing Jiancheng Bioengineering Institute, Nanjing, China). Lysozyme (LZM) activity (CK-E94755) was determined using ELISA kit (Shanghai Elisa Biotech Co., Ltd). The activity of α-amylase (C016-1-1), lipase (A054-2-1), and trypsin (A080-2-2) were determined with assay kits (Nanjing Jiancheng Bioengineering Institute, Nanjing, China). Total protein concentration (P0012) in the supernatant was determined using a Bicinchoninic Acid assay kit (Beyotime Biotechnology, Shanghai, China).

### RNA Extraction and Real-Time PCR

The total RNA in hepatopancreas and intestines was isolated using MolPure TRIeasy Plus Total RNA Kit (19221ES50; Yeasen Biotech Co., Ltd., Shanghai, China). The RNA concentration and quality were assessed with NanoDrop ND-2000 Spectrophotometer (Thermo Scientific, Waltham, MA, USA). The integrity of extracted RNA was determined by electrophoresis on a 1.2% (w/v) agarose gel. After that, 2,000 ng of RNA was reversely transcribed to cDNA in 20-μl reactions using Hifair^®^ III 1st Strand cDNA Synthesis SuperMix for qPCR (11141ES60; Yeasen Biotech Co., Ltd., Shanghai, China).

Then, real-time PCR was performed in a total 25 μl volume: 1 μl of cDNA template (≤50 ng); 1 μl of Forward primer (10 μM); 1 μl of Reverse primer (10 μM); 9.5 μl of DEPC-treated water (Sangon Biotech, Shanghai, China); and 12.5 μl of TB Green™ Premix EX Taq II™ (RR820 A, Takara Biotech, Dalian, China). A two-step real-time PCR amplification program was used: 95°C for 2 min and then 40 cycles of 95°C for 10 s and 60°C for 30 s.

Specific primers for target genes and housekeeping genes, designed on National Center for Biotechnology Information (NCBI), were synthesized by Sangon Biotech (Shanghai) Co., Ltd. (**Table S1**). All the real-time PCR analyses were performed using a quantitative thermal cycler (CFX96 Touch™ Real-Time PCR Detection System, Bio-Rad, USA). The gene expression levels were normalized using relative quantitative method (2^−ΔΔCT^) referencing the gene β-actin of shrimp (29).

### Intestinal DNA Extraction and Illumina Sequencing of 16S rRNA Genes

Genomic DNA sample was extracted from the intestine using the QiAamp^®^ PowerFecal^®^ Pro DNA Kit (Qiagen, Germany). To characterize bacterial community structures and compositions, the V4 region of 16S rRNA gene was amplified with the primer 515F/806R. Quality and purity of PCR products were assessed by Beijing Novogene Genomics Technology Co. Ltd. (China). Sequencing was conducted on an Illumina NovaSeq platform provided by Novogene Genomics Technology Co. Ltd. (Beijing, China). Paired-end reads were assigned to samples based on their unique barcode and truncated by cutting off the barcode and primer sequence and then merged using FLASH software (30, 31). The high-quality clean tags were obtained according to the QIIME (30, 31). The effective tags were finally obtained using UCHIME algorithm (32–35) and then clustered to the operational taxonomic units (OTUs) using UPARSE based on 97% sequence similarity (32–35). Representative sequence for each OTU was screened for further annotation using Silva Database (v132) based on RDP classifier. Taxonomic assignment was performed using RDP classifier based on the reference database (Greengenes database) (32–35). Alpha diversity analysis (observed species, Chao1, ACE, Simpson index, and Shannon index) and beta diversity on unweighted UniFrac for principal coordinates analysis (PCoA), non-metric multidimensional scaling (NMDS), and unweighted pair group method with arithmetic mean (UPGMA) clustering were calculated with QIIME and displayed with R software (v 3.6.2).

### High-Throughput Quantitative PCR

HT-qPCR approach was employed to investigate the profile of ARGs and mobile genetic elements (MGEs) in the intestine of shrimp (Magigene Technology Co. Ltd., Guangdong, China). A total of 383 primer sets were used to analyze antibiotic resistome, targeting major classes of ARGs (320 primer sets) and MGEs (63 primer sets). All DNA samples were diluted to 20 ng/μl using sterile water and amplified in triplicate for each primer set in a SmartChip Real-time PCR system. qPCR results were analyzed using SmartChip qPCR Software. Wells with multiple melting peaks or with amplification efficiencies beyond the range (0.8–1.2) were discarded. A threshold cycle (CT) of 31 was used as the detection limit, and only ARGs with amplification in all replicates were regarded as positive. Relative copy number was calculated using the following equation: relative gene copy number = 10^(31 − CT)/(10/3)^, where CT refers to quantitative PCR results. Roche was employed to obtain absolute 16S rRNA gene copies, which were used to calculate absolute copies number of ARGs and MGEs.

### Vibrio parahaemolyticus Challenge Test

After the feeding trial, the bacterial challenge test was carried out. Thirty shrimp were randomly selected from four tanks in each treatment, randomly separated into three groups and injected intramuscularly with 0.1 ml of *Vibrio parahaemolyticus* (5 × 10^6^ CFU/ml). The mortality in each replicate tank was recorded for 7 days.

### Statistical Analysis

Statistical software SPSS 22.0 for Windows (IBM SPSS Corporation, Chicago, USA) was used for the data analysis. Results were analyzed by one-way ANOVA. Tukey’s multiple-range test was used for the multiple comparisons of group means. Differences were regarded as significant when *p* < 0.05. Metastats analysis was performed to identify the bacterial taxa differentially represented between groups at genus or higher taxonomy levels (32–35). The Mann–Whitney test was used to compare the differences in ARGs and MGEs.

## Results

### Growth Performance

WGR and SGR of shrimp showed an increasing trend with the increase of MOS level, but no significant difference was observed when compared with the control group (M0) (*p* > 0.05). There was no significant difference in FI and FE among all groups (*p* > 0.05) (**Table 2**).

**Table 2:** Effects of dietary MOS on growth performance of Litopenaeus vannamei*.

Diets	M0	M2	M4	M8	M16
WGR (%)	292.70 ± 4.77	297.83 ± 13.78	305.47 ± 16.88	320.52 ± 13.47	319.68 ± 15.26
SGR (%/day)	2.44 ± 0.02	2.46 ± 0.06	2.50 ± 0.08	2.56 ± 0.06	2.56 ± 0.07
FI (%/day)	1.08 ± 0.02	1.09 ± 0.03	1.08 ± 0.05	1.05 ± 0.02	1.06 ± 0.05
FE	0.28 ± 0.01	0.28 ± 0.01	0.28 ± 0.02	0.30 ± 0.01	0.30 ± 0.02

IBW, initial body weight; FBW, final body weight; FI, feed intake; FE, feed efficiency; WGR, weight gain rate; SGR, specific growth rate; MOS, mannan oligosaccharide.

*Values are means ± SE, and values in a column not sharing the same superscript letter are significantly different (p < 0.05).

### Antioxidative Parameters

**Figure 1:**
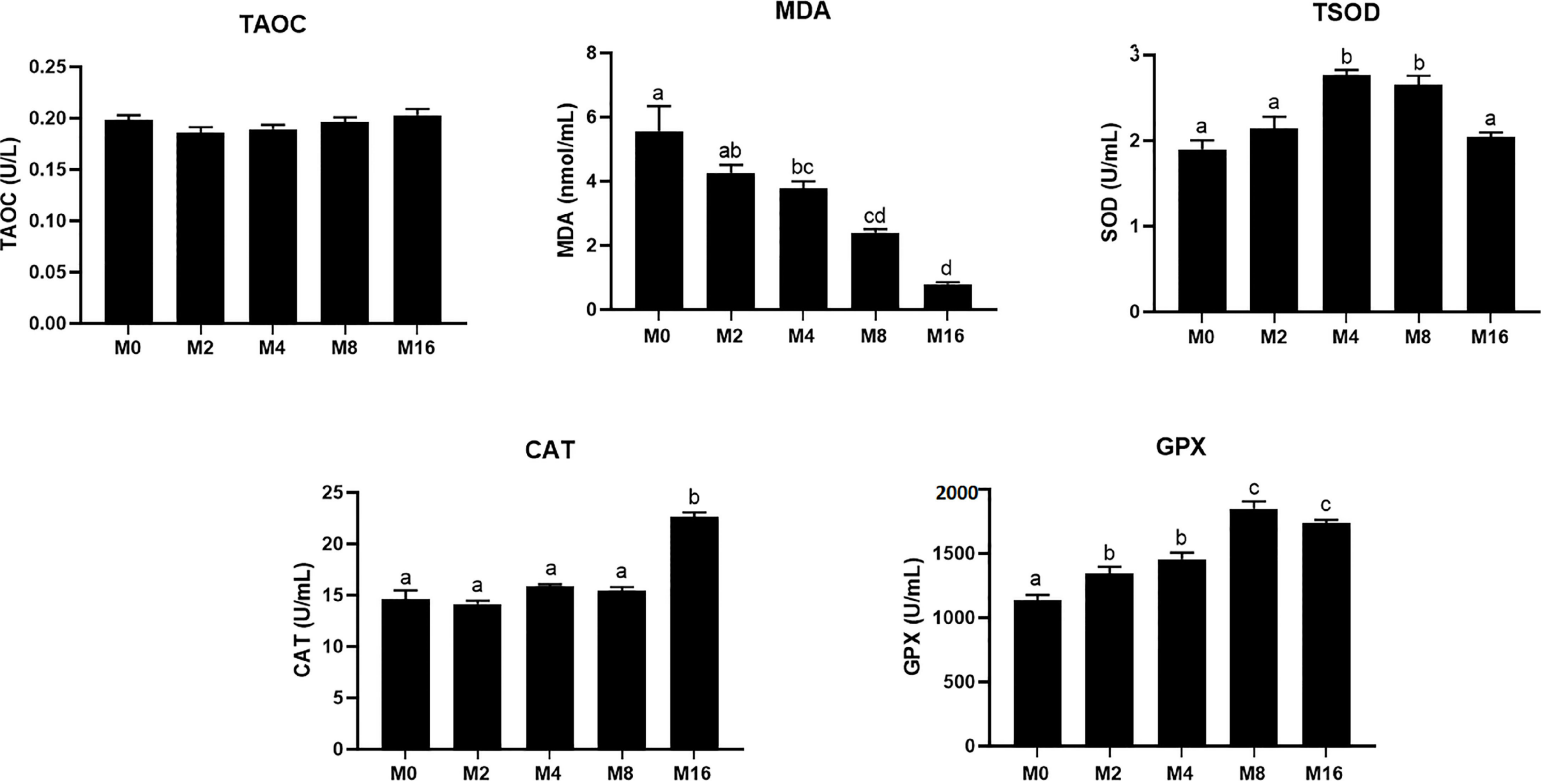
Effects of dietary MOS on antioxidative indices in plasma of *Litopenaeus vannamei*. ^a,b,c^ Value bars not sharing the same superscript letter are significantly different as evaluated by Tukey’s test (*p* < 0.05). T-AOC, total anti-oxidation capacity; TSOD, total superoxide dismutase; MDA, malondialdehyde; CAT, catalase; GPX, glutathione peroxidase; MOS, mannan oligosaccharide.

The MDA content in plasma was progressively decreased with the increase of dietary MOS, which had significantly lower values in M4, M8, and M16 compared with M0 (*p* < 0.05). The activity of TSOD increased initially and decreased afterwards with increasing dietary MOS levels, which was significantly higher in shrimp fed M4 and M8 compared with M0 (*p* < 0.05). Dietary MOS significantly enhanced the GPX activity in plasma (*p* < 0.05). The inclusion of 0.16% MOS in diet significantly increased the activity of CAT in plasma (*p* < 0.05). No significant difference was observed in T-AOC among all groups (**Figure 1**).

### Immunity

**Figure 2:**
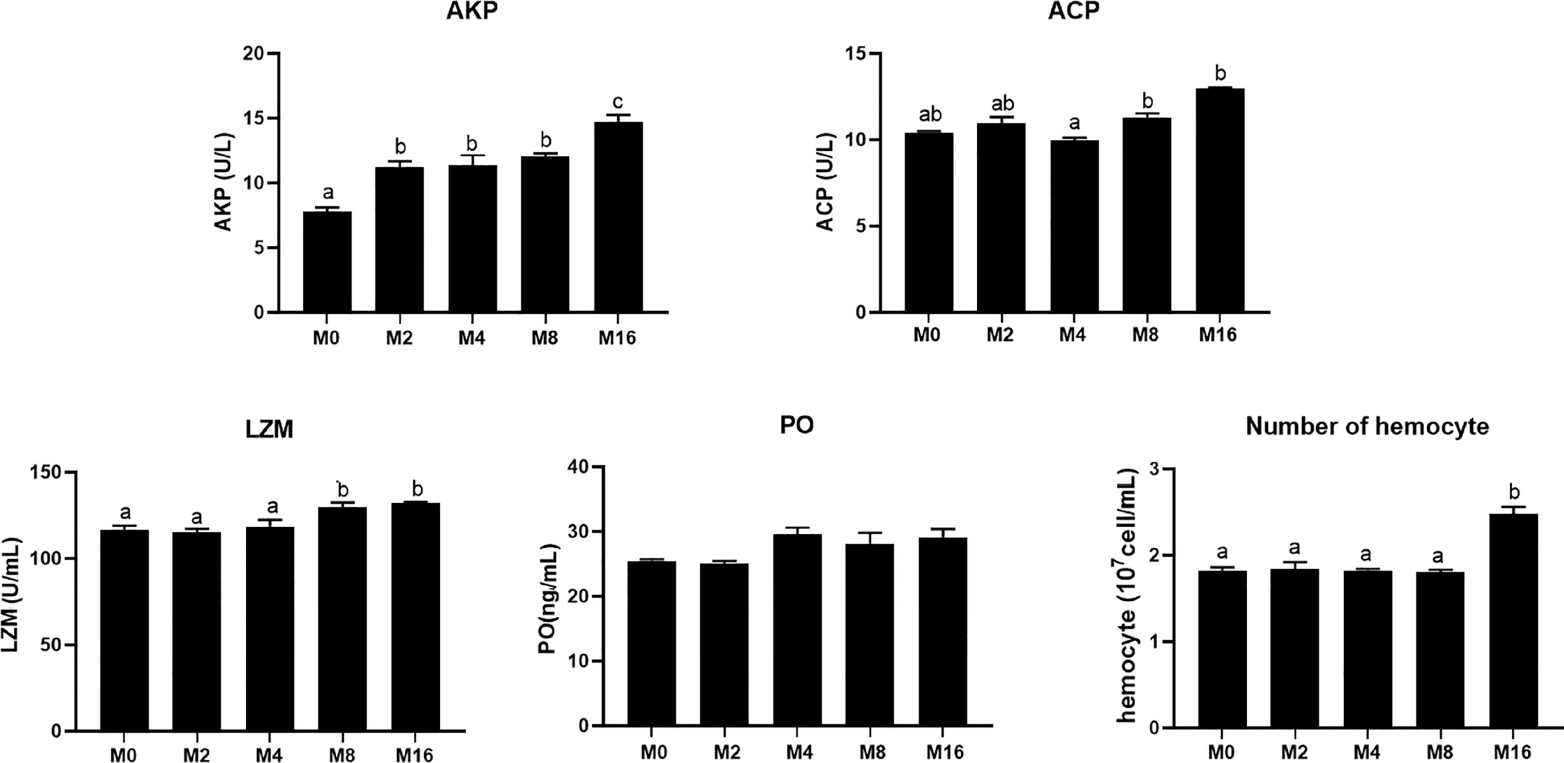
Effects of dietary MOS on non-specific immune indices in plasma and hemocyte counts of *Litopenaeus vannamei*. ^a,b,c^ Value bars not sharing the same superscript letter are significantly different as evaluated by Tukey’s test (*p* < 0.05). ACP, acid phosphatase; AKP, alkaline phosphatase; LZM, lysozyme; PO, phenol oxidase.

**Figure 3:**
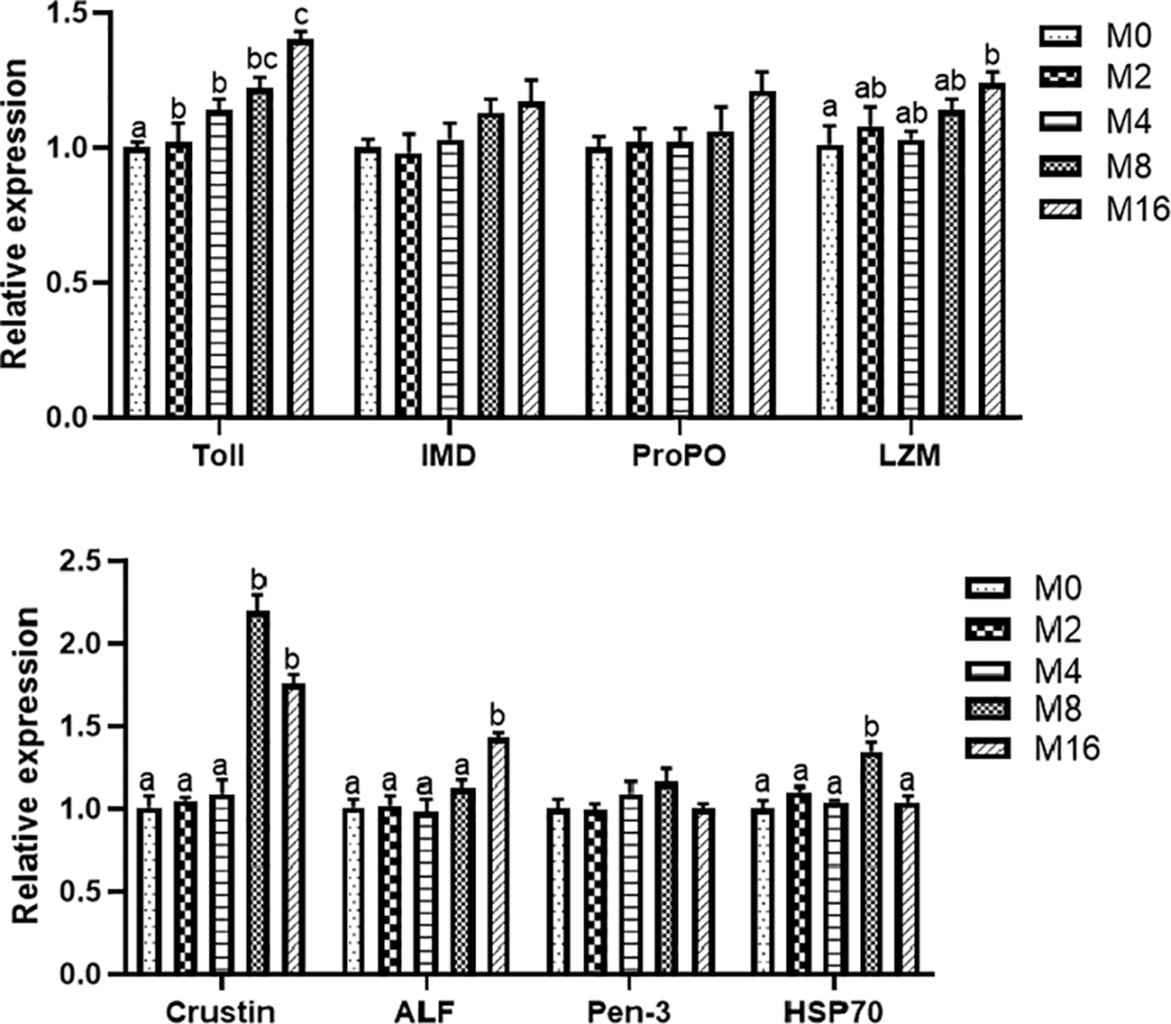
Effects of dietary MOS on expression of immune-related genes in hepatopancreas of *Litopenaeus vannamei*. ^a,b,c^ Value bars not sharing the same superscript letter are significantly different as evaluated by Tukey’s test (*p* < 0.05). IMD, immune deficiency; ProPO, pro-phenoloxidase; LZM, lysozyme; ALF, anti-lipopolysaccharide factor; Pen-3, penaeidins-3; HSP70, heat shock protein 70; MOS, mannan oligosaccharide.

Compared with the M0 diet, dietary MOS significantly enhanced the alkaline phosphatase (AKP) activity in plasma (*p* < 0.05). Higher levels of MOS (M8 and M16) significantly increased the LZM activity (*p* < 0.05). No significant difference was observed in acid phosphatase (ACP) and PO activities between MOS-supplemented groups and the control group (*p* > 0.05). The HC of shrimp fed M16 was higher compared with that fed M0 (*p* < 0.05) (**Figure 2**). The expression of Toll in the hepatopancreas was progressively elevated with the increase of dietary MOS (*p* < 0.05) (**Figure 3**). Diet M16 significantly upregulated the expression of LZM and ALF in the hepatopancreas (*p* < 0.05); diet M8 and M16 significantly upregulated the expression of Crustin (*p* < 0.05). The expression of HSP70 was significantly higher in shrimp fed M8 compared with M0 (*p* < 0.05). No significant difference was observed in the expression of immune deficiency (IMD), pro-phenoloxidase (ProPO), and penaeidins (Pen-3) between different groups (*p* > 0.05).

### Digestive Enzyme Activity

**Figure 4:**
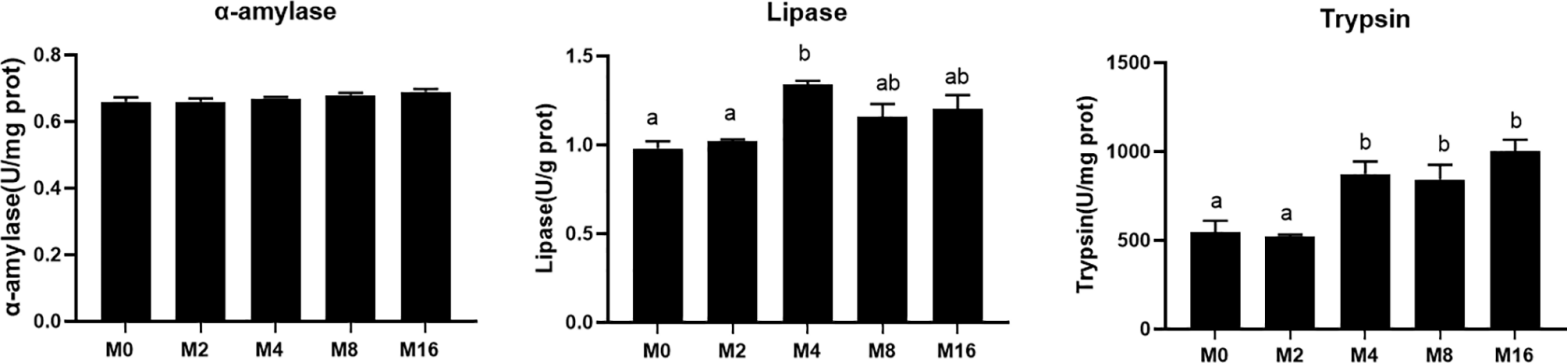
Effects of dietary MOS on digestive enzymes in hepatopancreas of *Litopenaeus vannamei*. ^a,b^ Value bars not sharing the same superscript letter are significantly different as evaluated by Tukey’s test (*p* < 0.05). MOS, mannan oligosaccharide.

The activity of digestive enzyme in hepatopancreas was significantly affected by the supplementation of MOS (**Figure 4**). The highest lipase activity was recorded in M4 (*p* < 0.05). M4, M8, and M16 significantly enhanced the activity of trypsin (*p* < 0.05). The activity of α-amylase was not affected by dietary MOS (*p* > 0.05).

### Intestinal Gene Expression

**Figure 5:**
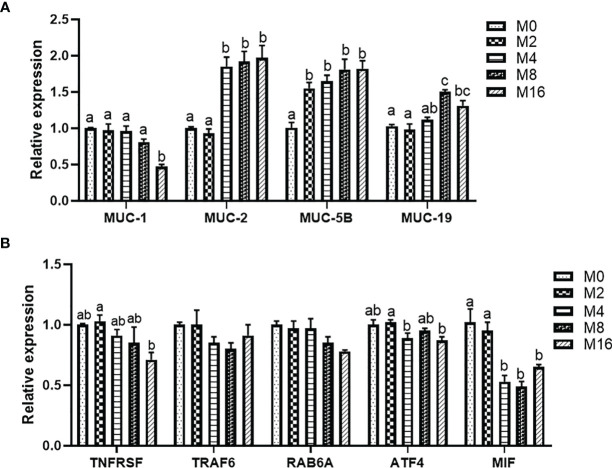
Effects of dietary MOS on the expression of mucin gene **(A)** and pro-inflammatory genes **(B)** in intestine of *Litopenaeus vannamei*. ^a,b,c^ Value bars not sharing THE same superscript letter are significantly different as evaluated by Tukey’s test (*p* < 0.05). MUC-1, mucin-1; MUC-2, mucin-2; MUC-5B, mucin-5B; MUC-19, mucin-19; TNFRSR, tumor necrosis factor receptor superfamily; TRAF6, tumor necrosis factor receptor-associated factor 6; RAB6A, Ras-associated protein 6A; ATF4, activating transcription factor 4; MIF, macrophage migration inhibitory factor; MOS, mannan oligosaccharide.

Diet M16 significantly downregulated the expression of intestinal MUC-1 (*p* < 0.05) (**Figure 5A**). Higher levels of MOS supplementation (M4, M8, and M16) significantly upregulated the expression of intestinal MUC-2 (*p* < 0.05). MOS supplementation significantly upregulated the expression of intestinal MUC-5B (*p* < 0.05). The expression of intestinal MUC-19 was significantly higher in the M8 and M16 groups compared with M0 (*p* < 0.05).

The results of intestinal pro-inflammatory gene expression showed that higher MOS levels (M4, M8, and M16) resulted in lower expression of MIF in the intestine (*p* < 0.05). A decreasing trend was observed in the expression of intestinal TNFRSF, TRAF6, RAB6A, and ATF4 of shrimp fed higher levels of MOS (M4, M8, and M16). However, no significant difference was observed in the MOS group when compared with the M0 group (*p* > 0.05) (**Figure 5B**).

### Intestinal Microbiota

The alpha diversity indices indicated that M8 led to higher richness of intestinal microbiota in terms of observed species, Chao1 index, and ACE index (*p* < 0.05). However, the addition of MOS did not affect the Shannon and Simpson indices (*p* > 0.05) (**Table 3**).

**Table 3:** Effects of dietary MOS on the alpha diversity of intestinal microbiota in *Litopenaeus vannamei*.

Diets	Observed species	Shannon	Simpson	Chao1	ACE
M0	195.00 ± 7.70^a^	2.39 ± 0.06	0.70 ± 0.02	214.54 ± 10.69^a^	211.38 ± 10.42^a^
M2	183.75 ± 15.99^a^	2.46 ± 0.13	0.73 ± 0.03	203.91 ± 15.05^a^	198.36 ± 16.05^a^
M4	231.50 ± 11.49^a^	2.34 ± 0.09	0.68 ± 0.03	259.73 ± 15.23^a^	252.67 ± 14.11^a^
M8	319.50 ± 28.50^b^	2.51 ± 0.18	0.67 ± 0.04	354.19 ± 27.68^b^	347.51 ± 27.68^b^
M16	228.75 ± 12.22^a^	2.63 ± 0.11	0.73 ± 0.03	260.16 ± 18.36^a^	245.32 ± 13.52^a^

*Values are means ± SE, and values in a column not sharing the same superscript letter are significantly different (p < 0.05).

**Figure 6:**
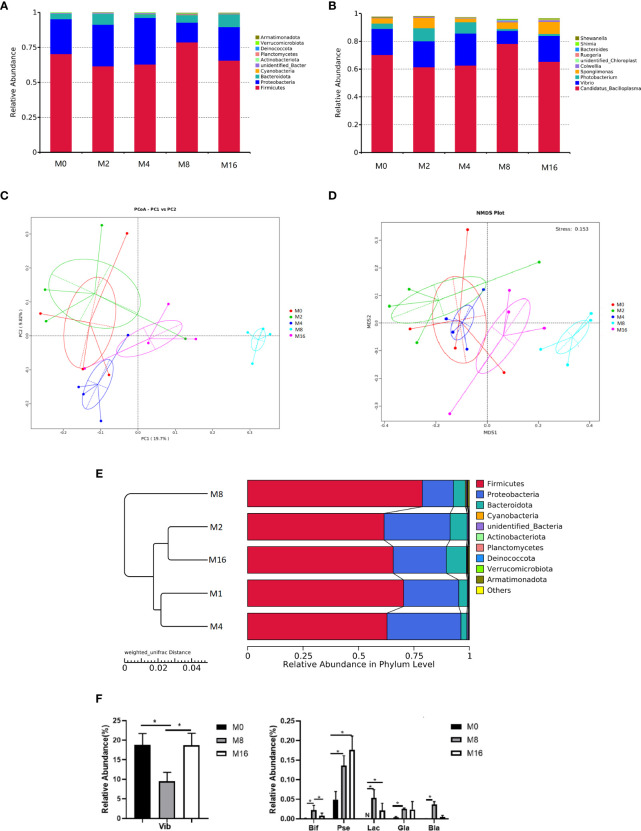
Effects of dietary MOS on the intestinal microbiota. Taxonomy classification of reads at the phylum **(A)** and genus **(B)** taxonomic levels. Only the top 10 most abundant (Based on relative abundance) bacterial phyla and genera are shown in the figures; other phyla and genera were all assigned as “Others.” Beta diversity of intestinal microbiota of *Litopenaeus vannamei*. **(C)** Principal coordinates analysis (PCoA). **(D)** Non-metric multidimensional scaling (NMDS), and **(E)** unweighted pair group method with arithmetic mean (UPGMA)-clustering trees based on unweighted UniFrac distance. **(F)** Some significantly changed abundance at genus level in M0, M8, and M16. *Significant difference between treatments (*p* < 0.05). Vib, *Vibrio*; Bid, *Bifidobacterium*; Pse, *Pseudoalteromonas*; Lac, *Lactobacillus*; Gla, *Glaciecola*; Bla, *Blautia*; MOS, mannan oligosaccharide.

At the phylum level, Firmicutes, Proteobacteria, and Bacteroidetes were detected as the predominant bacterial phyla in the intestine from all groups (**Figure 6A**). At the genus level, the bacterial composition of the M0, M2, and M4 groups were dominated by *Candidatus-Bacilloplasma*, *Vibrio*, *Photobacterium*, and *Spongiimonas*. However, *Candidatus-Bacilloplasma*, *Vibrio*, and *Spongiimonas* were the most abundant genera in the M8 and M16 groups (**Figure 6B**).

The PCoA, NMDS, and UPGMA-clustering tree were used to compare the similarity in the microbial community composition of shrimp fed different diets (**Figures 6C–E**). The results showed that as MOS level increased, samples clustered more distinctly according to the diets. Furthermore, a clear separation was observed between M8 and M0, indicating that dietary MOS had a strong effect on the overall structure of intestinal microbiota.

Some of the species that differed in the control (M0), 0.08% MOS (M8), and 0.16% MOS (M16) groups are shown in **Figure 6F**. M8 markedly increased the relative abundance of *Lactobacillus*, *Bifidobacterium*, *Pseudoalteromonas*, *Brautia*, and *Glaciecola* but significantly decreased the relative abundance of *Vibrio* (*p* < 0.05). Significant increase in the relative abundance of *Pseudoalteromonas* and *Lactobacillus* was also observed in the M16 group (*p* < 0.05).

### Detection of Antibiotic Resistance-Related Genes

By using HT-qPCR, a total of 40 ARGs and 11 MGEs were identified. These ARGs were classified based on the antibiotic to which they confer resistance, including aminoglycoside, beta-lactam, fluoroquinolone, glycopeptide, macrolide–lincosamide–streptogramin B (MLSB), multidrug, phenicol, sulfonamide, and tetracycline, with multidrug being the most abundant in all groups. Notably, there was a decline in the detected ARG abundance upon MOS supplementation at 0.08% (**Figure S1A**). In addition, the result of PCoA showed that the profile of ARGs in M8 was distinct from that in M0 (**Figure S1B**).

**Figure 7:**
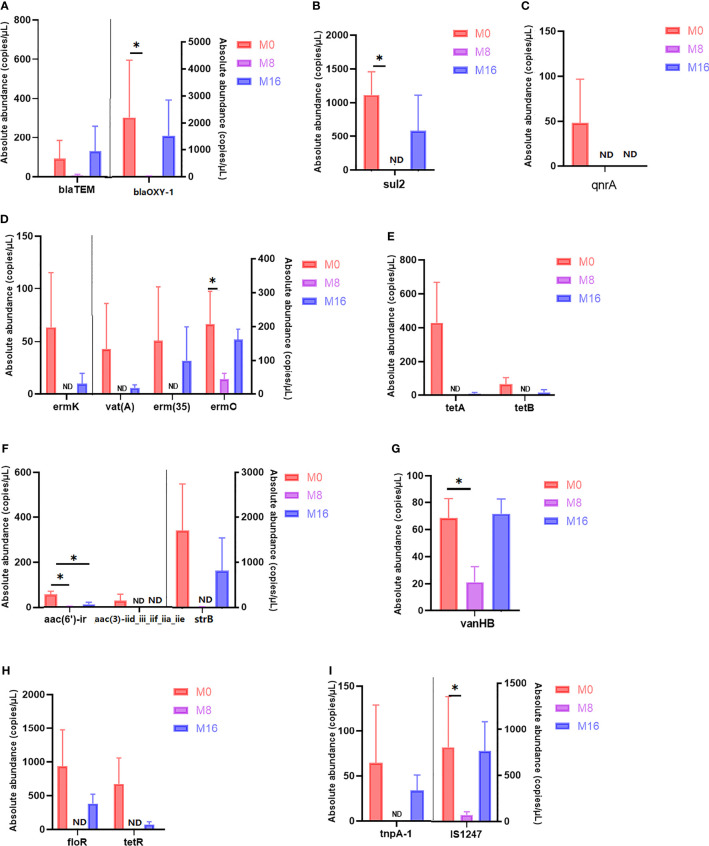
Quantity of antibiotic resistance genes in M0, M8, and M16. *Significant difference between treatments (*p* < 0.05). **(A)** Beta-lactam, **(B)** sulfonamide, **(C)** fluoroquinolone, **(D)** macrolide–lincosamide–streptogramin (MLSB), **(E)** tetracycline, **(F)** aminoglycoside, **(G)** glycopeptide, **(H)** multidrug, and **(I)** transposase. **(A–H)** ARGs; **(I)** MGEs. ARGs, antibiotic resistance genes; MGEs, mobile genetic elements.

Differential analysis of partial antibiotic resistance-related genes content is shown in **Figure 7**. M8 reduced the content of blaTEM and blaOXY-1, and significant differences were observed in blaOXY-1 (*p* < 0.05). No sul2 was detected in the M8 group, and a significant difference was observed when compared with the M0 group (*p* < 0.05). Qnra and aac (3)-iid_iii_iif_iia_iie were detected only in the M0 group, but not in the M8 and M16 groups. A lower content of ermK, vat(A), erm (35), tetA, tetB, strB, floR, and tetR was observed in the M16 groups than in the M0 group (*p* > 0.05), while it was not detected in the M8 group. The content of ermo and vanHB in the M8 group was significantly lower than that in M0 (*p* < 0.05). Compared with the control diet, dietary MOS at 0.08% and 0.16% significantly decreased the content of aac (6′)-ir (*p* < 0.05). A lower MGE content was also observed in MOS-supplemented group. IS1247 in the M8 group was significantly lower than that in the M0 group (*p* < 0.05), while tnpA-1 was not detected in the M8 group.

### Challenge Test

**Figure 8:**
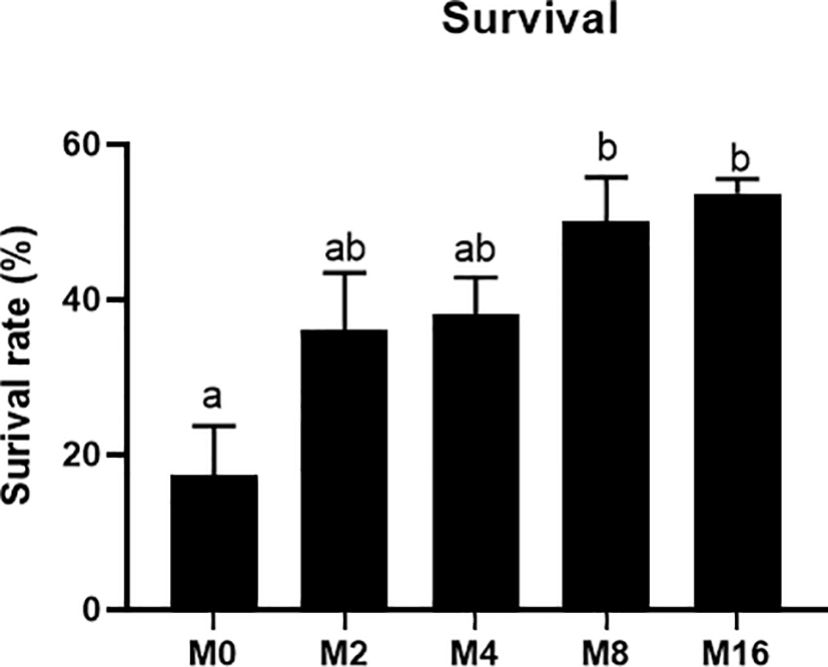
Effects of dietary MOS on mortality rate of *Litopenaeus vannamei* challenged with *Vibrio parahaemolyticus*. ^a,b^ Value bars not sharing the same superscript letter are significantly different as evaluated by Tukey’s test (*p* < 0.05). MOS, mannan oligosaccharide.

The results of *V. parahaemolyticus* challenge experiment showed that the mortality of shrimp fed M8 and M16 decreased by 32.59% and 36.29%, respectively, and significant difference was observed when compared with the M0 (*p* < 0.05) (**Figure 8**).

### Discussion

Previous studies have shown that MOS could promote the growth performance of broilers and poultry (36, 37). In aquatic animals, growth-stimulating effects of MOS in diets have also been observed. Torrecillas et al. (17) reported that the supplementation of MOS in diet increased the SGR of European sea bass. Studies in rainbow trout showed that dietary MOS at 0.15% significantly improved the growth performance (11). The growth-promoting effect of MOS has also been found in crustaceans, such as spiny lobster (*Panulirus homarus*) (38), green tiger prawn (*Penaeus semisulcatus*) (19), and Chinese mitten crabs (*Eriocheir sinensis*) (39). However, studies on Atlantic salmon (*Salmo salar*) (40) and gilthead sea bream (*Sparus aurata*) (12) did not find any growth-promoting effect of dietary MOS. This is consistent with the results of the present study. Although there was a tendency for dietary MOS to increase the WGR and SGR of shrimp, no significant difference was observed when compared with the control group. The inconsistent effect of MOS on growth performance may be related to the difference in species and development stage of animals, the environmental factors of aquaculture, cultivation time, and the difference of MOS dose.

MOS is immunogenic, which can stimulate the immune response and can bind to the surface of some toxins, viruses, and fungal cells, thus slowing down the absorption of antigens and enhancing the cellular and humoral immune responses of animals (41). Toll and IMD pathways are the major regulators of immune response in shrimp, which could regulate the expression of antimicrobial peptide (AMP) (20, 42). In the present study, dietary MOS significantly upregulated the expression of Toll gene in hepatopancreas, as well as the gene expression of ALF and Crustin, suggesting that MOS might regulate the level of AMPs by affecting the Toll pathway, which in turn promoted the immune response of shrimp. LZM is an important effector molecule in the innate immunity of shrimp, which could attack the peptidoglycan in bacterial cell walls and prevent the invasion of harmful bacteria (43). It has been shown that the addition of MOS to fish diets can increase the activity of LZM (18, 44). In line with the previous findings, dietary MOS enhanced the activity and gene expression of LZM in hepatopancreas. The vibriosis is a common disease in shrimp aquaculture, resulting in large numbers of shrimp deaths and significant economic losses to the shrimp industry (45). Although no significant difference was observed, there was an increasing tendency for the expression of IMD, ProPO, and Pen-3 in the MOS group. Considering these indicators together, we concluded that MOS promoted the immunity of shrimp organism. In this study, dietary MOS also significantly enhanced the resistance of shrimp to *V. parahaemolyticus*, which directly reflected the improvement of immune function by MOS.

The intestine represents the first line of host defense against pathogenic bacteria, where mucus immune complexes and microbial composition play an important role in intestinal function (46). Mucin, a major component of the mucus secreted by intestinal goblet cells to fight infection, contains mannosyl receptors, which could competitively bind to type 1 fimbriae of bacteria, thus assisting in clearance of pathogens (47). It was reported that dietary MOS increased the number of intestinal goblet cells and improved mucin production in broiler chickens (48, 49). In the present study, the supplementation of MOS in diets significantly increased the expression of intestinal MUC-2, MUC-5B, and MUC-19 but downregulated the expression of MUC-1, suggesting that MOS could regulate the intestinal immunity by affecting the secretion of mucins. Nevertheless, further studies are needed to investigate the precise role of different mucins in shrimp. Additionally, dietary MOS can downregulate the expression of MIF, a potential pro-inflammatory cytokine in vertebrates (50), which was considered to be important in innate immunity of shrimp (51).

The shrimp intestine harbors a diverse community of microbiota, which is of great importance for the host health (52). In the present study, dietary MOS led to higher richness of microbiota, which tended to be more favorable to intestinal microecology. Firmicutes, Proteobacteria, and Bacteroidetes were dominant in the intestines of shrimp in this study, which was consistent with the results of Huang et al. (53). There were some differences in the dominant bacteria at the genus level among different groups, with *Candidatus-Bacilloplasma*, *Vibrio*, and *Photobacterium* dominating in the control and lower-level MOS (M0, M2 and M4) groups, while *Candidatus-Bacilloplasma*, *Vibrio*, and *Spongiimonas* dominated in the higher-level MOS (M8 and M16) groups. Besides, more obvious clustering of microbiota in intestine was observed in M8, which was also clearly separated from the control. This indicated that dietary MOS at higher levels (M8 and M16) had a stronger effect on the overall profile of intestinal microbiota in the intestine of shrimp. Generally, MOS could reduce the colonization of pathogenic bacteria and enhance the growth of beneficial bacteria (54). The studies on livestock indicated that diet with MOS supplementation could lead to increase of *Lactobacillus* and *Bifidobacterium* (55) and decrease of *Escherichia coli* and *Salmonella* (55, 56) in the intestine. Similarly, the present results showed that dietary MOS significantly increased the abundance of *Lactobacillus* and *Bifidobacterium* and reduced the abundance of *Vibrio* in the intestine. Moreover, the abundance of *Pseudoalteromonas* and *Blautia* displayed a significant increase in MOS-supplemented groups. Bacteria CDM8 and CDA22 belonging to *Pseudoalteromonas* were identified as potential biocontrol agents against hepatopancreatic necrosis in shrimp culture and were able to reduce the abundance of *Vibrio* in the intestine (57). *Blautia* had a function of producing short-chain fatty acids that lowered intestinal pH and promoted resistance to intestinal colonization of pathogenic bacteria, thus in turn enhancing host health (58). The results indicated that dietary MOS was beneficial to improvement of intestinal bacteria community of shrimp.

In recent years, overuse or misuse of antibiotics in aquaculture has led to ARGs in the aquatic environment (59). ARGs, as emerging environmental contaminants, exist not only in the soil and water environment but also in animals (60). They are the root cause of bacterial resistance and increase the difficulty of disease control (61), as ARGs could be transferred horizontally to bacteria of the same or different genera by integration into MGEs (62). It has been reported that ARGs detected in in shrimp aquaculture were mainly sulfonamide, tetracycline, quinolone, and macrolide (63). In the present study, dietary MOS (M8 and M16) decreased the content of these resistance genes to some extent. TetA, tetB (tetracycline), ermk, erm (35), and vat (MLSB) were detected in the control group but not in the M8 group. A lower content of sul2 (sulfonamide) and ermo (MLSB) was observed in the M8 group compared with the control group. Qnra (quinolone) was not detected in the MOS group (M8 and M16) but in the control group. ARGs were the most common exogenous genes carried by transposons, which are important MGEs (64). ARGs could be transferred by transposons horizontally between strains, accelerating the transfer and spread of ARGs in the environment. We found that dietary MOS at 0.08% decreased the content of transposon IS1247. The data presented here demonstrated the ability of MOS to reduce the content and transfer of ARGs in shrimp intestine.

### Conclusion

The supplementation of MOS in diets significantly enhanced the antioxidation capacity and promoted the non-specific immunity and the resistance to *Vibrio* in shrimp. Furthermore, MOS could markedly improve the intestinal health, including the intestinal immunity and ecology. Especially, dietary MOS increased the abundance of potential probiotics such as *Bifidobacterium* and *Lactobacillus* and reduced the abundance of potential pathogen *Vibrio*. Notably, we found that MOS had the ability to reduce the content and transfer of ARGs. MOS could be an effective additive to promote the intestinal and organismal health of shrimp and mitigate the degree of antibiotic resistance, especially when added at 0.08%–0.16% in the diets. We also found that some parameters were better when MOS was supplemented at 0.08% than 0.16%. Therefore, we need to further investigate whether the addition of MOS at levels higher than 0.16% will have adverse effects on shrimp.

## Data Availability Statement

The data presented in the study are deposited in http://ncbi.nlm.nih.gov/sra, under the accession number PRJNA764415.

## Ethics Statement

The animal study was reviewed and approved by the Animal Care Committee of Ocean University of China.

## Author Contributions

Conceptualization: YZ, KM, and GL. Methodology: YZ, TW, and JY. Formal analysis: YZ, TW, and JY. Investigation: TW and JY. Data curation: TW and JY. Writing—original draft preparation: TW and YZ. Writing—review and editing: YZ, TW, GL, ML, and RZ. Supervision: YZ and KM. Project administration: KM and YZ. Funding acquisition: KM and YZ. All authors contributed to the article and approved the submitted version.

## Funding

This work was financially supported by the National Key R&D Program of China (2019YFD0900104), the National Natural Science Foundation of China (No. 31872577), and China Agriculture Researches System (Grant No. CARS 47-G10).

## Conflict of Interest

Author RZ was employed by company Beijing Alltech Biological Products (China) Co., Ltd.

The remaining authors declare that the research was conducted in the absence of any commercial or financial relationships that could be construed as a potential conflict of interest.

## Publisher’s Note

All claims expressed in this article are solely those of the authors and do not necessarily represent those of their affiliated organizations, or those of the publisher, the editors and the reviewers. Any product that may be evaluated in this article, or claim that may be made by its manufacturer, is not guaranteed or endorsed by the publisher.
