# Corrigendum: Effects of dietary mannan oligosaccharides on non-specific immunity, intestinal health, and antibiotic resistance genes in Pacific White Shrimp *Litopenaeus vannamei*

**DOI:** 10.3389/fimmu.2022.1015734

**Published:** 2022-09-12

**Authors:** Tiantian Wang, Jinzhu Yang, Gang Lin, Mingzhu Li, Ronghua Zhu, Yanjiao Zhang, Kangsen Mai

**Affiliations:** ^1^ The Key Laboratory of Aquaculture Nutrition and Feed (Ministry of Agriculture), The Key Laboratory of Mariculture (Ministry of Education), Ocean University of China, Qingdao, China; ^2^ Institute of Quality Standards and Testing Technology for Agricultural Products, Chinese Academy of Agricultural Sciences, Beijing, China; ^3^ College of Agriculture, Ludong University, Yantai, China; ^4^ Beijing Alltech Biological Products (China) Co., Ltd., Beijing, China

**Keywords:** mannan oligosaccharide, immunity, intestinal microbiota, antibiotic resistance genes, *Litopenaeus vannamei*

In the published article, there was an error in the MOS concentration description. In the **Conclusion** section, instead of “We also found that some parameters were better when MOS was supplemented at 0.016% than 0.08%” it should be “We also found that some parameters were better when MOS was supplemented at 0.08% than 0.16%”.

The authors apologize for this error and state that this does not change the scientific conclusions of the article in any way. The original article has been updated.

## Publisher’s note

All claims expressed in this article are solely those of the authors and do not necessarily represent those of their affiliated organizations, or those of the publisher, the editors and the reviewers. Any product that may be evaluated in this article, or claim that may be made by its manufacturer, is not guaranteed or endorsed by the publisher.

