# Supplementary material for: Effects of Dietary Mannan Oligosaccharides on Non-Specific Immunity, Intestinal Health, and Antibiotic Resistance Genes in Pacific White Shrimp Litopenaeus vannamei
Source: Front Immunol. 2021 Nov 24;12:772570. doi: 10.3389/fimmu.2021.772570 (PMC8652215; doi:10.3389/fimmu.2021.772570)
Supplement: Supplementary file 1 [file DataSheet_1.docx]

## Effects of dietary mannan oligosaccharides on non-specific immunity, intestinal health and antibiotic resistance genes in Pacific white shrimp *Litopenaeus vannamei*

Tiantian Wang ^1^, Jinzhu Yang ^1^, Gang Lin ^2^, Mingzhu Li ^3^, Ronghua Zhu ^4^, Yanjiao Zhang ^1,^ * and Kangsen Mai ^1^

^1^ The Key Laboratory of Aquaculture Nutrition and Feed (Ministry of Agriculture), the Key Laboratory of Mariculture (Ministry of Education), Ocean University of China, Qingdao 266003, China

^2^ Institute of Quality Standards and Testing Technology for Agricultural Products, Chinese Academy of Agricultural Sciences, Beijing 100081, China

^3^ College of Agriculture, Ludong University, Yantai 264025, China

^4^ Beijing Alltech Biological Products (China) Co., Ltd., Beijing 100600, China

***** **Correspondence**: yanjiaozhang@ouc.edu.cn (Y.Z.); Tel.: +86-532-8203-

**TABLE S1** Primers used in quantitative real-time PCR.

| Gene | Forward (5’→3’) | Annealing temperature (℃) | GenBank no. |
| --- | --- | --- | --- |
| Crustin | F: AGTAGGTGTTGGTGGTGGTTTC | 60 | AY488497 |
|  | R: AGTCGCTTGTGCCAGTTCC |  |  |
| ALF | F: TACAAGGGGAAAATGTGGTGC | 60 | GQ227486 |
|  | R: TGAAGGCTTTCTCGACGAAGT |  |  |
| Pen-3 | F: TAAGCCACCCAGGCAGAATA | 60 | AJ310694.1 |
|  | R: TAAGCCACCCAGGCAGAATA |  |  |
| HSP70 | F: AGGAGACCGCTGAGGCTTAC | 60 | AY645906.1 |
|  | R: CAGCACATTCAGACCCGAGAT |  |  |
| Toll | F: CTGGACTCCCTCACAAACCTT | 60 | DQ923424 |
|  | R: GCCCACGCGATTTCCTATA |  |  |
| IMD | F: TGGGTCCGTGTCCAGTGAT | 60 | FJ592176.1 |
|  | R: GCCAATGTGAACCGCAGAG |  |  |
| ProPO | F: GAACTCCATTCCGTCCGTCTG | 60 | AY723296.1 |
|  | R: TAAAGGTACGTGGGCAGGTC |  |  |
| LZM | F: GGCTCGGAAGTTGGCGATGATG | 60 | AY170126.2 |
|  | R: CGATGGCTCAATGGCGAAGAGG |  |  |
| MUC-1 | F: TGCCAGCCACGTCCTCCTTG | 60 | Duan et al., 2018 |
|  | R: CCGCAGCCGAGGCAGTCC |  |  |
| MUC-2 | F: CTTGACGCATACGCTCAGGTTCC | 60 | Duan et al., 2018 |
|  | R: TCCGCCGCCTTCATCCTCTG |  |  |
| MUC-5B | F: GAAGAGGAGGAAGAGGACGAGGAG | 60 | Duan et al., 2018 |
|  | R: GGACCACCAGGCACAAGAACATC |  |  |
| MUC-19 | F: GGCTCGGAAGTTGGCGATGATG | 60 | Duan et al., 2018 |
|  | R: CGATGGCTCAATGGCGAAGAGG |  |  |
| TNFRSF | F: GACGAACCCTGTTTCCTGGT | 60 | JN180641.1 |
|  | R: TCGGCTGCTGTTATTCGCTT |  |  |
| TRAF6 | F: CAGCTGCGGCTTGAAGTTTT | 60 | HM581680 |
|  | R: GTTCTACGACGAAGCCGTGA |  |  |
| RAB6a | F: CAGTGTATGAAGCCAGCAGAG | 60 | JX073679.1 |
|  | R: GGTCGTATTTGTCCTCATTAACTC |  |  |
| ATF4 | F: AACACAGGAAGCAGCAGAAC | 60 | JX908828.1 |
|  | R: ACGGCAGTGATGGTCTCTC |  |  |
| MIF | F: AACACAGGAAGCAGCAGAAC | 60 | KC513658.1 |
|  | R: ACGGCAGTGATGGTCTCTC |  |  |
| β-actin | F: GAAGTAGCCGCCCTGGTTGT | 60 | AF300705.2 |
|  | R: GGATACCTCGCTTGCTCTGG |  |  |

Abbreviations：ALF, anti-lipopolysaccharide factor; Pen-3, penaeidins-3; HSP70, heat shock protein 70; IMD, immune deficiency; ProPO, pro-phenoloxidase; LZM, lysozyme; MUC-1, mucin-1; MUC-2, mucin-2; MUC-5B, mucin-5B; MUC-19, mucin-19; TNFRSR, tumor necrosis factor receptor superfamily; TRAF6, tumor necrosis factor receptor-associated factor 6; RAB6A, ras-associated protein 6A; ATF4, activating transcription factor 4; MIF, macrophage migration inhibitory factor.


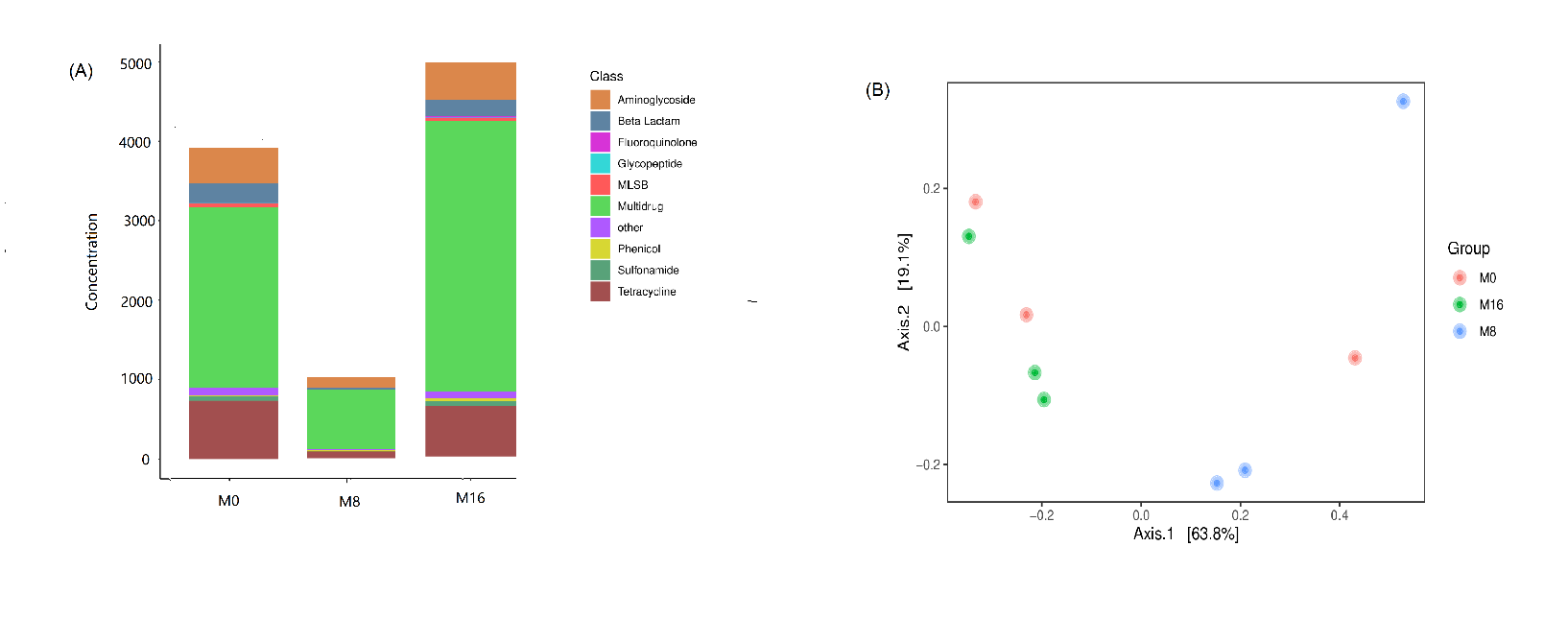


**FIGURE S1** Abundance and diversity of antibiotic resistance genes. (A) Detected number of ARGs. Samples were classified based on the antibiotic to which they confer resistance. (B) Principal coordinate analysis (PCoA) based on the Bray-Curtis distance showing the distict distribution pattern of ARGs.
